# Gene Expression Profiles of Beta-Cell Enriched Tissue Obtained by Laser Capture Microdissection from Subjects with Type 2 Diabetes

**DOI:** 10.1371/journal.pone.0011499

**Published:** 2010-07-13

**Authors:** Lorella Marselli, Jeffrey Thorne, Sonika Dahiya, Dennis C. Sgroi, Arun Sharma, Susan Bonner-Weir, Piero Marchetti, Gordon C. Weir

**Affiliations:** 1 Section on Islet Transplantation and Cell Biology, Research Division, Joslin Diabetes Center and the Department of Medicine, Harvard Medical School, Boston, Massachusetts, United States of America; 2 Molecular Pathology Unit, Massachusetts General Hospital, Harvard Medical School, Boston, Massachusetts, United States of America; 3 Section of Endocrinology and Metabolism of Organ Transplantation, Department of Endocrinology and Metabolism, University of Pisa, Pisa, Italy; Uppsala University, Sweden

## Abstract

**Background:**

Changes in gene expression in pancreatic beta-cells from type 2 diabetes (T2D) should provide insights into their abnormal insulin secretion and turnover.

**Methodology/Principal Findings:**

Frozen sections were obtained from cadaver pancreases of 10 control and 10 T2D human subjects. Beta-cell enriched samples were obtained by laser capture microdissection (LCM). RNA was extracted, amplified and subjected to microarray analysis. Further analysis was performed with DNA-Chip Analyzer (dChip) and Gene Set Enrichment Analysis (GSEA) software. There were changes in expression of genes linked to glucotoxicity. Evidence of oxidative stress was provided by upregulation of several metallothionein genes. There were few changes in the major genes associated with cell cycle, apoptosis or endoplasmic reticulum stress. There was differential expression of genes associated with pancreatic regeneration, most notably upregulation of members of the regenerating islet gene (REG) family and metalloproteinase 7 (MMP7). Some of the genes found in GWAS studies to be related to T2D were also found to be differentially expressed. IGF2BP2, TSPAN8, and HNF1B (TCF2) were upregulated while JAZF1 and SLC30A8 were downregulated.

**Conclusions/Significance:**

This study made possible by LCM has identified many novel changes in gene expression that enhance understanding of the pathogenesis of T2D.

## Introduction

Pancreatic beta-cells play a central role in the development and progression of type 2 diabetes (T2D) [1]. In subjects predisposed to T2D beta-cell dysfunction occurs early in the course of the disease [1], [2], with deterioration occurring during the transition from the “normal” state to impaired glucose tolerance and then to frank diabetes [1], [3]. Beta-cell dysfunction is now accepted to be associated with reduced beta-cell mass [4], [5], which has even been shown to be present in the state of impaired glucose tolerance [4]. Beta–cell abnormalities likely result from a combination of genetic and environmental factors. This reduction in beta-cell mass could be caused by either inadequate birth of beta-cells, increased death by apoptosis or necrosis, or some combination of the two [6]. The development of secretory dysfunction is closely tied to rising glucose levels and presumed to be caused by the process of glucotoxicity [1].

Genome-wide association studies and single gene studies have identified polymorphisms within genes potentially influencing the beta–cell development and function [7]. However, the characteristics of gene expression in beta-cells of T2D subjects, which determines the cell phenotype, are largely unknown; only a few studies have evaluated the gene expression of islets isolated from T2D subjects [8]–[15]. A limitation of these studies is that they have been performed on isolated human islet preparations, which contain substantial numbers of islet non-beta cells, duct cells, and acinar cells. In addition, the islets have been studied after the trauma of the isolation procedure that causes changes in gene expression [16]–[18]. These limitations can be overcome by studying beta–cell enriched tissue dissected from the pancreatic tissue directly, using the laser capture microdissection (LCM) technique [18], [19].

In the present study we performed the LCM on ten control and ten T2D subjects; messenger RNA was analyzed by microarray and real-time PCR was performed on selected genes. The results showed a variety of alterations in gene expression of beta–cells obtained from T2D donors.

## Results and Discussion

### Clinical characteristics

Pancreas specimens from ten non-diabetic and ten T2D subjects were studied. Their clinical characteristics can be found in Table 1.

**Table 1:** Clinical characteristics of type 2 diabetic and control subjects.

	Controls	Type 2 diabetic donors
Gender (males/females)	6/4	7/3
Age (years)	60±5	67±7
BMI (Kg/m^2^)	30.5±6.5	30.9±6.2 (n = 9)
Cause of death	8 CVD, 1 TR, 1 SA	8 CVD, 1 TR
Known diabetes duration (years)	-	5.3±2.3 (n = 7)
Anti-diabetes therapy	-	Oral (n = 6); insulin (n = 3)

All donors were White. Data are expressed as mean ± standard deviation (SD). The two groups were different for age (p = 0.019) and comparable for BMI (p = 0.917). The duration of diabetes was 5.3±2.3 years (n = 7, data of 3 diabetic donors are not available); three diabetic subjects were insulin treated and six received oral anti-diabetic therapy, information related to the anti-diabetic treatment of one subject is not available.

CVD: cardiovascular disease; TR:trauma; SA: surgical accident.

### Approach to LCM and islet anatomy

It is important to point out that LCM was used selectively in that islets were chosen that had relatively well defined patches of beta-cells with endogenous immunofluorescence. It was important to do this because human islets are known have clumps of α and δ cells in the islet core [6], which means that great care must be taken with LCM to avoid the non beta-cells. Thus, there are some populations of beta-cells that cannot be collected for geographical reasons. We expect that these selected beta-cells are representative of beta-cells throughout the pancreas but must be cautious about this assumption. It cannot be excluded that beta-cells in islets of different size and locations may be subjected to different paracrine and environmental influences that could alter gene expression. Another point is that the endogenous immunofluorescence of the beta-cells may emanate from lipofuscin, which is thought to accumulate as beta-cells age [20]. Thus, it is possible that the beta-cells collected with LCM are older than the overall population. However they would be comparable in the two groups since no differences in the intensity of immunofluoresence could be discerned between beta-cells of control versus T2D pancreases.

The 10 T2D pancreases had the range of pathology expected for T2D. Amyloid could be found in some islets in most of the pancreases, as was expected from previous observations [21], [22]. Moreover, much islet variation was seen, which included islets with fibrosis, islets with high non-beta-/beta-cell ratios, and a range of islet size. Extracellular lipid deposits were not particularly noticeable. We did not stain for alpha-cells, so we do not know the alpha-cell/beta-cell ratio specifically, but we had the impression that non-beta-cell/beta-cell ratio is up, as is well described by many including MacLean and Ogilvie in 1955 [23] and then more recently by Clark et al [24] and Yoon et al [25]. As was also done for the control pancreases, relatively well defined patches of beta cells were chosen for LCM. Thus, for the T2D pancreases, this means that care was taken to avoid islets with amyloid and/or fibrosis.

### Expression profiles in samples obtained from T2D donors compared to control donors

**Figure 1:**
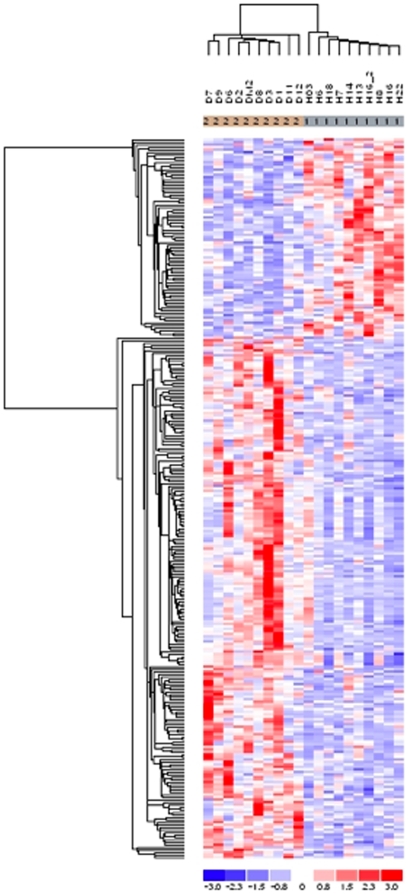
Hierarchical clustering of differentially expressed genes. Hierarchical clustering analysis of genes differentially expressed was performed using dChip software. The distances among high-dimensional expression profiles are represented as a dendrogram that arranges the clustered samples in terms of similarity to one another. The analysis showed a clear separation in two groups of samples from diabetic and control subjects. The analysis divides samples into groups with similar patterns in gene expression data (a p value of less than 0.05 was considered).

By using the lower confidence bound (LCB) parameter at the cutoff of 1.2, DNA-Chip Analyzer (dChip) software identified 2,062 differentially expressed probe sets, corresponding to 1,920 transcripts. Among them, 1,203 transcripts were upregulated and 717 were downregulated in samples obtained from T2D subjects as compared to non-diabetic controls. Using the p value parameter, 7,164 probe sets, corresponding to 6,384 transcripts were differentially expressed at p<0.05 (3,464 transcripts upregulated and 2,920 downregulated), whereas 2,133 probe sets, corresponding to 1,870 transcripts were different at p<0.01 (1006 upregulated and 864 downregulated). Hierarchical clustering analysis of differentially expressed genes showed a clear separation between diabetic and non-diabetic samples (Fig. 1).

**Figure 2:**
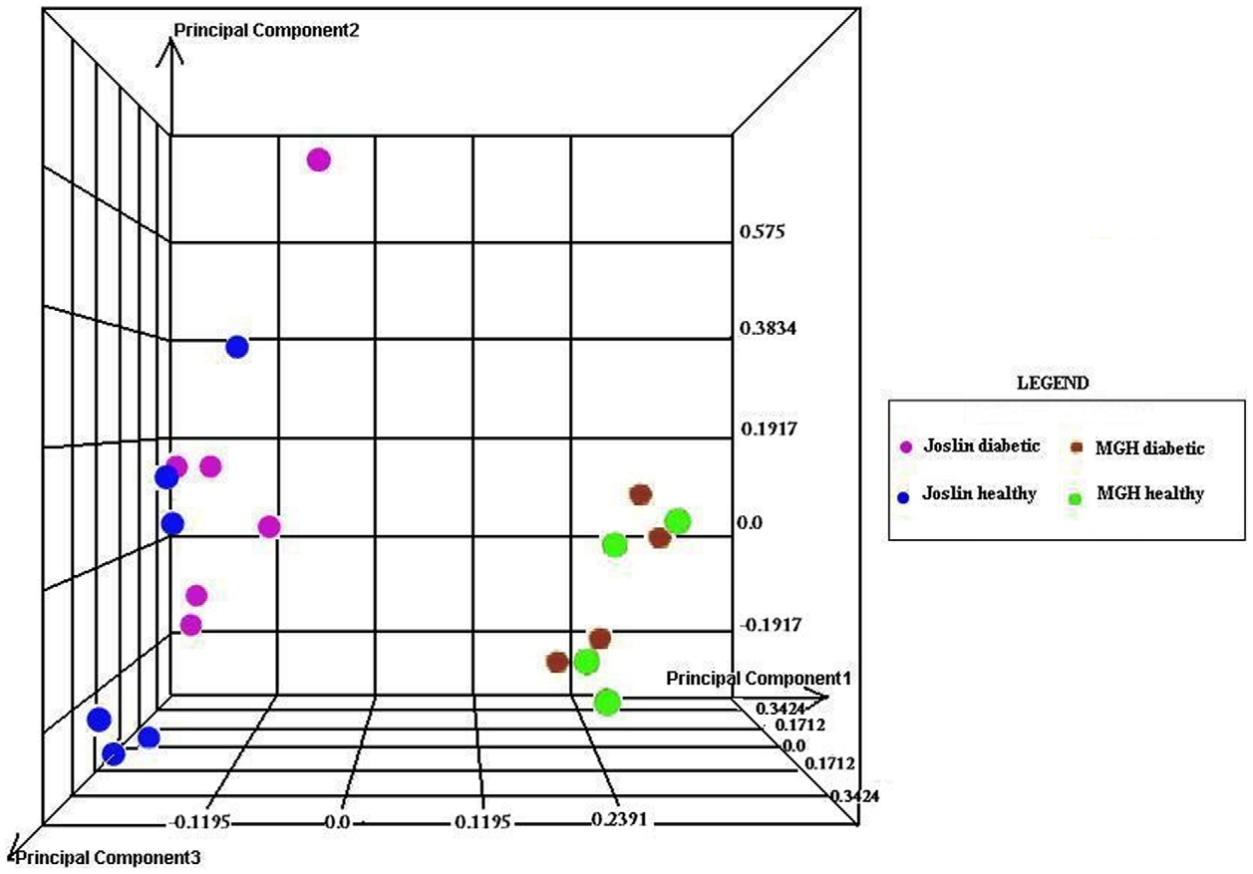
Principal Component Analysis. Principal Component Analysis (PCA) reduces the number of variables and sort microarray experiments into groups. The analysis was performed using Rosetta Resolver software, version 7.2.2.0, with all the array data after normalization by dChip software. Three principal components were generated and plotted; each individual point identifies a single expression profile. The first principal component (PC1) that captures the maximum amount of variation between samples determined the clustering of samples into two groups: one group (12 samples, left) was run at the Joslin Diabetes Center Genomic Core facility, the other group (8 samples, right) was run at the Genomic Core facility of the Massachusetts General Hospital. Variations were also observed along the second principal component (PC2) and the third principal component (PC3). The purple and brown dots refer to samples from diabetic subjects run at the Joslin Diabetes Center and Massachusetts General Hospital, respectively; the blue and green dots refer to control subject samples run at the Joslin Diabetes Center and Massachusetts General Hospital.

As reported in Figure 2, the Principal Component Analysis showed that samples run at the Genomic Core facilities of the Joslin Diabetes Center and the Massachusetts General Hospital, at two different times, clustered in two different groups. All data are MIAME compliant and the row data have been deposited in a MIAME compliant database (GEO, accession numbers: GSE20966).

### Approach to analysis

Most of the analysis was done with dChip software. We first looked at groups of genes thought to be important for the beta-cell abnormalities known to occur in T2D. They were grouped into categories of function. Other genes were included because they seemed to be good candidates to be important; some were differentially expressed, others were not. Clusters of genes with common function were assessed with GSEA. In assessing the signal strength of arrays as determined by dChip, the strength can be a general indicator of the abundance of the mRNA, but caution must be used for interpretation. Two major variables: the amplification process will change the abundance of the transcripts and low signal strength may results from inefficient probes. We know that some of the high strength signals are incorrect. For example, the array signal strength for insulin and glucagon was similarly high, yet when unamplified mRNA obtained with LCM was assessed with quantitative PCR, the insulin transcript levels were far higher than glucagon [18]. Nonetheless, the signals are very consistent for comparing groups such as samples from T2D versus controls. Moreover, the general strength of the signals provides insights into the relative strength of various isoforms. For example creatine kinase, brain (CKB), which may be important for glucose stimulated insulin secretion (GSIS), was highly expressed while the muscle form (CKM) was hardly expressed at all (Table S1).

### Islet hormones and receptors

The array signals for insulin (INS), glucagon (GCG), somatostatin (SST) and IAPP were all very high and only IAPP was differentially expressed, downwards (Table S2). These signals clearly do not provide accurate information about the actual abundance of message, as we have shown previously using PCR on samples collected by LCM [18]. It seems this is particularly a problem when the message is very highly expressed. Nonetheless, the reduced expression of IAPP is not surprising because similar reduction has been found in rodents with diabetes, which is probably a result of glucose toxicity [26]. No differential expression was found for either the receptors for glucagon (GCGR) or GLP1 (GLP1R); the signals for the gastric inhibitory polypeptide receptor (GIPR) were very weak.

### Islet transcription factors

In our previous studies with the rat partial pancreatectomy model, with multiplex PCR on isolated islets we found downregulation of pdx1, nkx6.1, pax6, neurod and hnf1alpha [26]. In the present study there was down regulation of HNF1A (TCF1) but no change in the expression of NKX6-1 or NEUROD1 (Table S2). PAX6 was upregulated. Unexpectedly, PDX1 was upregulated but the signal strength was low compared to other transcription factors. This might be due to low amplification efficiency of PDX1 transcript or inefficient binding of the transcript to the corresponding probe set. Other transcription factors not changed included NKX2-2, PAX4, HNF4A, neurogenin 3 (NEUROG3), and MAFB. PAX6 and ARX, which are normally found in alpha-cells [27], were upregulated, which raises the question as to whether the beta-cells of T2D developed some alpha-cell characteristics or whether the LCM of the T2D tissue somehow collected more alpha-cell tissue. It is possible that an increase in the ratio of alpha-cells to beta-cells might have made it more likely that a few more alpha-cells were contained in the material captured from the T2D pancreases. However, great care was taken to restrict the LCM to fluorescent beta-cells. If there were some inadvertent inclusions, one might have expected variability of PAX6 and ARX expression among the T2D pancreases, but the consistency was similar to that seen in the controls. Another possibility is that beta-cells in T2D express alpha-cell genes more highly. Others and we have found that some single beta-cells obtained by flow cytometry have a low level of glucagon gene expression [28], [29]. This becomes especially interesting with the recent report by the group of Herrera that alpha-cells may be able to transdifferentiate into beta-cells during regeneration [30].

### Glucose metabolism

Because beta-cells in a diabetic environment have profoundly abnormal GSIS, components of glucose metabolism pathways that are linked to secretion can be expected to be abnormal in T2D. The expression results of noteworthy genes are shown in Table S1. With regard to glucose transport, GLUT2 was downregulated in the T2D sample while GLUT1 was unchanged. This is of interest because while GLUT2 is thought to be important for rodents, evidence suggests that GLUT1 is largely responsible for glucose transport in human beta-cells [31]. For the glycolytic enzymes, no change was seen in the expression of glucokinase (GCK). Three forms of phosphofructokinase (PFKM, P and L) were highly but not differentially expressed. Aldolase B (ALDOB) was highly expressed and upregulated while enolase 1, alpha (ENO1) was modestly expressed and downregulated. Lactate dehydrogenase A (LDHA), which normally has very low expression in beta-cells [32] was upregulated. Pyruvate carboxylase (PC), which is highly expressed in rodent islets [26], [33], had low signal strength and no differential expression.

For gluconeogenesis, which is thought to be negligible in normal beta-cells, phosphoenolpyruvate carboxykinase 1 (PCK1) was upregulated, while the Fructose-1, 6-bisphosphatase (FBP2) and glucose-6-phosphatase (G6PC and G6PC3) enzymes genes had low signal strength and no differential changes. The mitochondrial enzyme dihydrolipoamide dehydrogenase (DLD), which is the E3 component of pyruvate dehydrogenase complex, was downregulated. Of enzymes of the tricarboxylic acid cycle, the only potentially interesting changes were a modest reduction in aconitase 2 (ACO2) and an increase of mitochondrial succinate dehydrogenase complex, subunit C (SDHC). There was no differential expression of the uncoupling protein genes 1, 2, or 3. Notable changes in mitochondrial shuttle genes were down regulation of both mitochondrial glycerol-3-phosphate dehydrogenase 2 (GPD2) and cytosolic malic enzyme 1 (ME1). ATP citrate lyase (ACLY), a cytosolic gene important for the production of acetyl CoA was clearly downregulated. These enzymes are thought to be important for GSIS [12], [34].

MacDonald et al recently reported reduced expression of GPD2 and ACLY in isolated islets from subjects with T2D [12], which agrees with our findings. They also found reductions in isocitrate dehydrogenase 1 (NADP+), soluble (IDH1), propionyl Coenzyme A carboxylase, beta polypeptide (PCCB), malate dehydrogenase 2, NAD (mitochondrial, MDH2) and 3-oxoacid CoA transferase 1 (OXCT1). None of these changes were confirmed in our study.

These data may provide some clues to the mechanisms responsible for glucotoxicity. We have extensively studied changes of islet gene expression 4 wk after 90% pancreatectomy in rats and found marked changes in glucose metabolism genes [26], [35]–[37]. The general trend was that genes highly expressed in beta-cells such as GLUT2 and GCK were downregulated while normally suppressed genes such as LDHA were upregulated. There was only some agreement between these and the present data. The combined rodent and human data provide a valuable partial picture, but the ideal study remains to be done to better understand beta-cells in T2D. Results obtained with isolated islets from cadaver donors have their limitations. LCM is an important advance, but the amplification step brings potential artifact and arrays of the future will provide better information. Other weaknesses of the present approach are the serious illness of the donors prior to death and the pancreases being subjected to hours of cold ischemia time. We tried to exclude a history of premorbid hyperglycemia in the nondiabetic controls, but with the use of steroids, and the presence of brain damage and stress, there may been periods of hyperglycemia that could have caused some glucotoxicity changes. A goal for the future should be to obtain pancreatic tissue from well-characterized patients at surgery. Frozen sections can be subjected to LCM, and enough RNA should be obtained to avoid the amplification step.

### Lipid metabolism

With regard to lipid metabolism (Table S1), fatty acid synthase (FASN) expression was modestly upregulated. It is interesting that L-3-hydroxyacyl-Coenzyme A dehydrogenase (HADH), short chain, which is part of the beta oxidation pathway in mitochondria, was downregulated because mutations in this gene have been linked to hyperinsulinemic hypoglycemia [38]. Another downregulated gene of potential interest was stearoyl-CoA desaturase (SCD), which contrasts with the increase found in Zucker fatty (ZF) rats after partial pancreatectomy [39].

### Channels and adrenergic receptor

In the evaluation of channels important for GSIS (Table S1), KIR6.2 (KCNJ11) had weak signal strength and no differential expression, but SUR1 was highly expressed and downregulated in diabetes. The Calcium channel, voltage-dependent, L type, alpha 1D subunit (CACNA1D) was also downregulated. Inositol 1,4,5-triphosphate receptor, type 3 (ITPR3), which could be important for inducing calcium release, was downregulated. Potassium voltage-gated channel, KQT-like subfamily, member 1 (KCNQ1), which acts to repolarize cells, was clearly upregulated in the samples from diabetic subjects. Another change that could influence secretion is downregulation of the adrenergic, alpha-2A-, receptor (ADRA2A) that mediates the adrenergic suppression of insulin secretion [40].

### Exocytosis

Reports of downregulation of exocytosis molecules and gene expression in islets from human T2D and GK rats, include syntaxin-1A, SNAP-25, VAMP-2, nSec1 (Munc18), Munc 13-1, synaptotagmin V and synaptophysin [11]. However, in our study (Table S2), syntaxin 1A (STX1A), synaptobrevin 2 (VAMP2), cellubrevin (VAMP3), Synaptophysin (SYP) and Syntaxin binding protein 1 (STXBP1, Munc18-1) had high signal strength but no differential expression. Synaptobrevin 1 (VAMP1) had much lower signal strength. Synaptosomal-associated protein, 25 kDa (SNAP25) was modestly downregulated and Unc-13 homolog B (C. elegans) (UNC13B) was clearly downregulated. Synaptotagmin V (SYT5) had little signal strength but Synaptotagmin IV (SYT4) was highly expressed. Both N-ethylmaleimide-sensitive factor (NSF) and N-ethylmaleimide-sensitive factor attachment protein, alpha SNAP (NAPA) were highly, but not differentially, expressed.

### Insulin signaling

In contrast to the findings of Gunton et al that were performed with isolated islet preparations of variable purity from subjects with T2D [8], we found increased expression of the insulin receptor (INSR) and the insulin-like growth factor receptor (IGF1R) in the beta-cell tissue from T2D (Table S1). Increased expression was also found for inositol polyphosphate-5-phosphatase (INPP5D, SHIP1) and inositol polyphosphate phosphatase-like 1 (INPPL1, SHIP2). Other upregulated genes included v-Ha-ras Harvey rat sarcoma viral oncogene homolog (HRAS) and v-raf-1 murine leukemia viral oncogene homolog 1 (RAF1). Of note, Forkhead box 01 (FOXO1) was downregulated. The AKTs, 1, 2 and 3 all had strong but no differential expression.

### ARNT, ARNT2 and HNF-1alpha

A major decrease in the expression of aryl hydrocarbon receptor nuclear translocator (ARNT) was reported in isolated islets of T2D subjects by Gunton et al [8]. This decrease was accompanied by reductions in HIF 1 alpha (HNF1A), the insulin receptor (INSR), several glycolytic enzymes including glucose-6-phospate isomerase (GPI), phosphofructokinase (PFK), aldolase (ALDO), and phosphoglucomutase (PGM). We had good to excellent signal strength for all of these genes but found no differential expression, except for an increase in ALDOB (Table S1). Gunton et al also reported a decrease in HNF4 alpha, but our signal strength for this gene was too low to be meaningful.

### Cell cycle genes

There were few changes of significance in the expression of cell cycle genes (Table S2). Of the cyclins, cyclin D2 (CCND2) and D3 (CCND3) were the most highly expressed, but there was no differential expression. Of the cyclin-dependent kinases, CDK4 and CDK6 were more highly expressed than CDK2. Of the cell cycle inhibitors, only two tended to be upregulated, p16 (INK4a), p<0.06, and p21 (Cip1). There were no changes of the E2F family transcription factors. Retinoblastoma 1 (RB1) and RBL2: retinoblastoma-like 2 (RBL2, p130) were well expressed but not differentially. The signal for retinoblastoma-like 1 (RBL1, p107) was very weak. Of interest, the CDK5 regulatory subunit associated proteins (CDK5RAP) 1, 2 and 3 were all differentially upregulated. They have been associated with neuronal development and spindle check point function [41]. Because so much of cell cycle control is exerted at the posttranslational level, these few modest changes in gene expression must not be over interpreted. The suggestion of change for p16 and p21 need to followed up; p16 in particular appears to be an important marker of aged cells that have restricted capacity for regeneration [42]. There has been interest in the epigenetic control of replication [43], but there was no differential expression of the following candidate genes EZH2, MLL, PRC1, JMJD3, or BMI1.

### ER stress

The ER stress genes were notable for their lack of change (Table S3). The few changes of interest included some mannosidases; isoforms MAN1B1 and MAN2B2 were upregulated. ER degradation enhancer, mannosidase alpha-like 3 (EDEM3) was downregulated. Endoplasmic oxidoreductin-1-like protein B (ERO1LB) was highly expressed and downregulated. Endoplasmic reticulum protein 27 kDa (ERP27), a noncatalytic member of the protein disulfide isomerase, was modestly upregulated as were DnaJ (Hsp40) homolog, subfamily C, member 3 (DNAJC3) and protein disulfide isomerase family A, member 4 (PDIA4).

Other genes that were highly expressed and had no change included: X-box binding protein 1 (XPB1), activating transcription factor 6 (ATF6), eukaryotic translation initiation factor 2A (EIF2A), UDP-glucose ceramide glucosyltransferase-like 2 (UGCGL2), DNA-damage-inducible transcript 3 (DDIT3, CHOP), heat shock 70 kDa protein 5 (HSPA5, BIP), heat shock protein 90 kDa beta (Grp94), member 1 (HSP90B1), and mitogen-activated protein kinase 8 (MAPK8, JNK). Wolfram syndrome 1 (WFS1), the loss of which leads to apoptosis of beta cells through ER stress [44], was highly, but not differentially, expressed.

There has been great interest in the possibility that ER stress contributes to the loss of beta-cells in T2D, and data indicate that parts of the process are activated in beta-cells of people with T2D [45]–[47]. One must be cautious about interpreting data from isolated islets because of the trauma of isolation, which could influence the expression of ER stress genes. It is possible that our own data was influenced by severe premorbid illness of the cadaver donors. One must remember that the unfolded protein response of ER stress has a number of features that protect cells from excessive demand for folding [48], so it is possible that the few changes we found were beneficial.

### Apoptosis

There was no differential expression of the major genes implicated in apoptosis (Table S3). These include the anti-apoptotic genes B-cell CLL/lymphoma 2 (BCL2) and BCLX, and the pro-apoptotic genes BAD, BAK1, BAX and BIM. FAS had very low signal strength and was upregulated with the probe set (g4507582_3p_a_at) covering the 10%, and recognizing the 3′ end of the transcript. Likewise, no changes were found for caspases 3, 6 or 7.

With regard to other death mechanisms, genes found upregulated in a study using isolated human islets exposed to cytokines were BIRC3, BCL2A1, TNFAIP3, CFLAR [49] – most were unchanged in ours but other interesting gene changes were found. In particular, survivin (BIRC5) was modestly upregulated, although with a less specific probe set. Survivin enhances proliferation and survival of cancer cells and seems to have similar effects upon beta-cells [50]. Other upregulated genes that could be important are TRAF3, TRADD and TRAF7. Interestingly, BOK and TRAF3IP2 were downregulated.

### Oxidative stress

Expression of some key genes related to oxidative stress are shown in Table S3. The antioxidant thioredoxin (TXN) was highly expressed and downregulated. The mitochondrial thioredoxin 2 (TXN2), which regulates mitochondrial membrane potential and protects against oxidant-induced apoptosis [51] was upregulated with a probe set with weaker signals but not with a probe set with stronger signals. Thioredoxin interacting protein (TXNIP), which in models of diabetes is overexpressed and seems to have deleterious effects on beta-cells [52], was strongly but not differentially expressed. Likewise, thioredoxin reductase 1 (TXNRD1), which may enhance ROS generation [53], was not differentially expressed.

Other genes thought to be important for protection against oxidative stress [54] were not differentially regulated including the glutathione peroxidases 3 and 4 (GPX3,GPX4) and catalase. Heme oxygenase 1 (HMOX1) was not different and had a weak signal. The mitochondrial superoxide dismutase 2 (SOD2) was upregulated; the extracellular form of superoxide dismutase 3 (SOD3) was as well but less significantly.

There were notable changes in the expression of a variety of metallothionein genes with a number being clearly upregulated and others tending in that direction. These include the metallothioneins 1E, 1G, 1M, 1X and 2A. In addition, metallothionein 1 pseudogene 2 was upregulated. Metallothioneins are involved in the regulation of metal metabolism, especially zinc, which is important for insulin biosynthesis and storage, and they have potent antioxidant effects because their cysteine residues can capture harmful oxidant radicals [55]. Their expression can be increased with oxidative stress.

Another interesting highly expressed downregulated gene is protein phosphatase 1E (PPM1E, PP2C domain containing), which is a member of a family implicated as negative regulators of cell stress response pathways and cell growth [56]. In summary, these data, taken in their entirety, suggest that beta-cells in T2D are chronically subjected to oxidative stress. Other changes of interest were up regulation of GADD45 variants, which contribute to DNA repair and are upregulated by the stress of growth arrest.

### Inflammation

Data on inflammation from this study have been reported in earlier publications [57], [58].

### Regeneration

**Figure 3:**
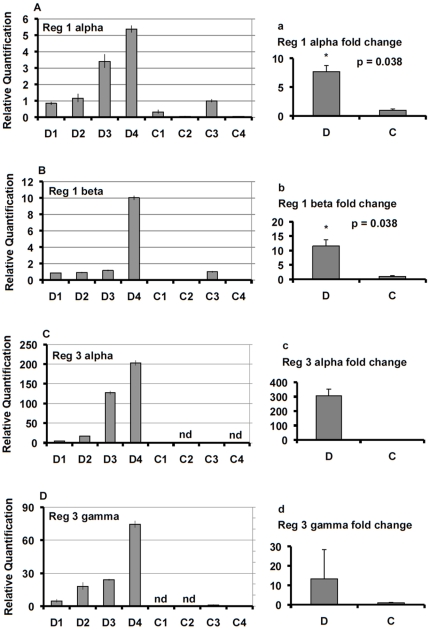
Real-time PCR analysis of the regenerating islet genes Reg 1 alpha, Reg 1 beta, Reg 3 alpha, and Reg 3 gamma. The assay was performed on samples from four T2D subjects (D) and four controls (C). Panels A–D show the relative gene expression of each single sample. In panels a–d data are reported as mean ± SE ratios of relative expression values from T2D samples (D) over values from control samples (C). The statistical significance was evaluated by the two-tailed Student's t-test using the dCt values (not performed with Reg 3 alpha and Reg 3 gamma since the expression was not detectable in two control samples each).

**Figure 4:**
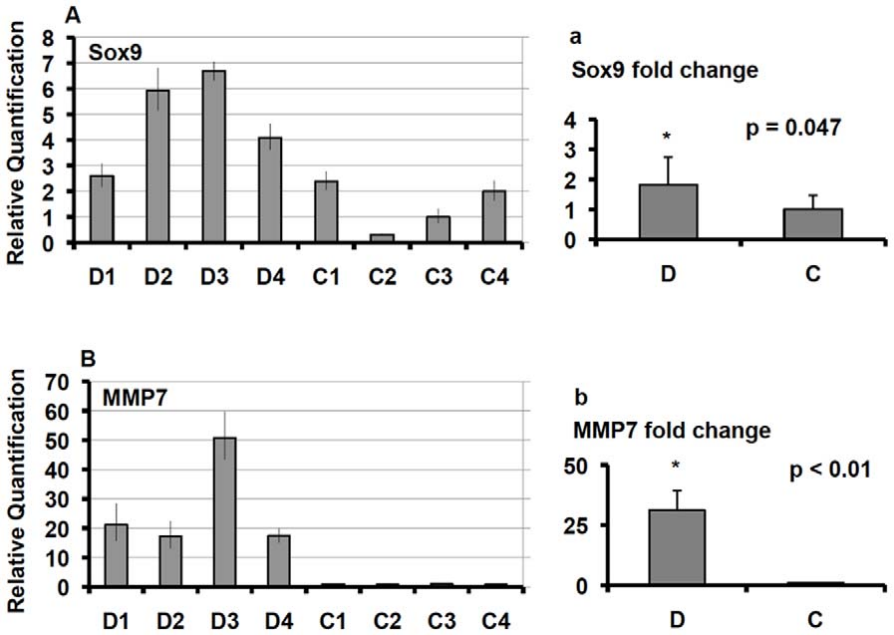
Real-time PCR analysis of Sox9 and matrix metalloproteinase 7 (MMP7) genes. The assay was performed on samples from four T2D subjects (D) and four controls (C). Panels A–B show the relative gene expression of each single sample. In panels a–b data are reported as mean ± SE ratios of relative expression values from T2D samples (D) over values from control samples (C). The statistical significance was evaluated by the two-tailed Student's t-test using the dCt values.

A variety of genes potentially involved in pancreatic islet development and regeneration were evaluated (Table S3). Genes belonging to the regenerating islet gene (REG) family were upregulated in samples of T2D subjects (Table S3); among them, REG1A and REG3G were those with the highest differential expression, and REG3G was the gene with the highest expression variability among diabetic subjects (Table S3). The expression of REG1A, REG1B, REG3A, and REG3G was quantified by real-time PCR, performed on samples from four T2D subjects and four controls, which, confirmed the differential expression between the two groups (Fig. 3 A–D). The roles of Reg family of proteins continue to be intriguing but poorly understood. Reg was first found in the islets after partial pancreatectomy in rats [59]. Reg1 has been implicated in beta-cell growth by association and by administration of the peptide [60], and a knock-out resulted in reduced islet mass [61]. Reg has also been found in beta-cells in mice with new onset type 1 diabetes [62]. Reg3A (HIP/PAP) is expressed in multiple tissues and has been associated with liver regeneration; it has been found to have mitogenic and anti-apoptotic functions [63]. The peptides human proIslet peptide (HIP) and islet neogenesis associated protein (INGAP) are related to sequences in REG3A, and are candidates for inducing neogenesis [64]. We also found that the transcription factor SOX9 was upregulated in samples from T2D donors (Table S3), and this differential expression of SOX9 was confirmed by real-time PCR (Fig. 4 A). This finding is of interest because SOX9 is known to be present in pancreatic duct cells and has been implicated in islet development by maintaining pancreatic progenitors [65]. The metalloproteinase MMP7 was upregulated in samples of T2D subjects (Table S3). The real-time PCR performed in samples obtained from four T2D subjects and four controls confirmed the differential expression of MMP7 between the two groups (Fig. 4 B). MMP-7 is involved in development, cancer invasion and sending epithelial signals to stromal cells [66], [67].

### Other growth and development genes

Because of indications of a regeneration process taking place, WNT signaling genes were examined (Table S2). Most of the WNT genes had no differential regulation except for down regulation of WNT6. Of interest, dishevelled (dsh homolog 2, DVL2), which is linked to WNT signaling and expressed in some cancers [68], was upregulated. In addition, dickkopf homologs (DKK) 3 and 4, which are important for development [69], were upregulated. Another upregulated gene was sulfatase 1 (SULF1), which is important for development and survival [69]. Frizzled homolog 4 (FZD4) was downregulated. Secreted frizzled-related proteins (SFRP) 2 and 5 were upregulated. We have previously found increases of SFRP expression in differentiating human pancreatic duct cells in vitro [70]. There was no differential expression of transcription factor 7-like 2 (TCF7L2).

Notch signaling is important for differentiation of pancreatic precursor cells to islet cells [27]. There was no differential expression of notch 1, 2 and 3; notch 3 was most highly expressed (Table S2). There was, however, a large differential increase of notch homolog 2 (Drosophila) N-terminal like (NOTCH2NL). No differential expression was found with delta-like (DLL) 1, 3 or 4, nor of delta-like 1 homolog (DLK1, pref1), but this was very highly expressed. Jagged 1 (JAG1) was not differentially expressed, but jagged 2 (JAG2) was upregulated. Hairy and enhancer of split 1 (HES1) showed no difference, nor did presenilins (PSEN) 1 and 2, or MFNG O-fucosylpeptide 3-beta-N-acetylglucosaminyltransferase (MFNG, manic fringe).

A variety of other genes have been suggested to be involved in islet development [70]. There was downregulation of SMAD3 and SMAD5, which are downstream targets of TGF beta. There was modest upregulation of BMP1, isoform 3, and BMP5. Other downregulated genes included cellular retinoic acid binding protein 1 (CRABP1) and ubiquitin carboxyl-terminal esterase L (UCHL) 1 and 3. BAMBI, which functions as a negative regulator of TGF-beta signaling during development [71], was upregulated. Inhibin, beta E (INHBE), a member of the activin beta family, is thought to be involved in pancreatic exocrine cell growth and proliferation [72] and was upregulated as was LASS1, a member of the TGF-beta superfamily, involved in cell growth and differentiation [73].

Endothelin 3 (EDN3) was very highly expressed but markedly downregulated in T2D, while the expression of its receptor (EDNRB) was unchanged. This secreted factor was found to enhance proliferation and survival of intestinal goblet cells [74]. Ephrin-A3 (EFNA3) was upregulated, which is of interest because this tyrosine kinase, has been found play a role in development and also influence insulin secretion [75].

### Tumor suppressors

We wondered if there were a regeneration process that was held in check by increased expression of tumor suppressors (Table S2). However, there was no differential expression of RB1, TP53, PTEN or APC. Other genes have been found to suppress metastases including MED23, DRG1, CD81, NME 1 and 2, TIMP2 and BRCA1; none were differentially changed.

### Genes associated with type 2 diabetes

Recent genome wide association studies (GWAS) have identified a number of gene loci associated with type 2 diabetes, many of which are related to beta-cell function or development [7]. As shown in Table 2, the genes with upregulated expression include: IGF2BP2, TSPAN8, and HNF1B (TCF2). Downregulated expression was found for JAZF1 and SLC30A8. Then there were genes with clear signal strength and little to no suggestion of differential expression: CDKAL1, TCF7L2, CDKN2A/2B, FTO, WFS1, THADA, ADAMTS9, HHEX, IDE, and CDC123. Finally there were genes with little or no expression: CAMK1d NOTCH2, KCNJ11, MC4R, and PPARG. While these data are unique, it is not clear whether the genes with differential expression have pathophysiological importance. Some of the gene polymorphisms must cause problems with function and development, but there is no particular reason to have expected their level of expression to be altered in this group of non-genotyped subjects.

**Table 2:** Gene expression of molecules associated with type 2 diabetes according to genome wide association studies (7).

Probe ID	Gene symbol	Ctrl	T2D	LCB	p value
**Genes associated with Type 2 Diabetes**					
**g5729883_3p_at**	**IGF2BP2**	**53±6**	**79±8**	**1.2**	**0.017**
**g4759237_3p_at**	**TSPAN8**	**67±8**	**101±11**	**1.2**	**0.026**
**g4507396_3p_at**	**HNF1B**	**102±5**	**123±5**	**1.1**	**0.011**
**Hs.5437.2.A2_3p_at**	**JAZF1**	**122±13**	**76±7**	**−1.3**	**0.008**
**Hs.170042.0.A1_3p_at**	**SLC30A8**	**3608±223**	**2818±118**	**−1.1**	**0.007**
214877_3p_at	CDKAL1	88±3	85±5	−0.9	0.670
Hs.173638.0.S3_3p_at	TCF7L2	1111±72	1112±77	0.9	0.995
g4502748_3p_at	CDKN2A	317±32	426±44	1.1	0.060
g11386206_3p_a_at	CDKN2B	25±2	26±2	0.9	0.686
g1710215_3p_at	FTO	2429±61	2494±58	1.0	0.448
g13376995_3p_at	WFS1	981±75	1145±90	1.0	0.178
4864673C_3p_s_at	THADA	354±18	391±19	1.0	0.167
Hs.16450.0.A1_3p_at	ADAMTS9	24±6	29±7	0.6	0.587
g10835016_3p_at	HHEX	42±11	41±8	−0.5	0.933
Hs.1508.0.S2_3p_at	IDE	80±7	67±5	−1.0	0.184
g5174422_3p_at	CDC123	68±7	63±5	−0.9	0.577
Hs.47883.0.A1_3p_at	CAMK1D	20±2	21±3	0.8	0.585
g11275977_3p_a_at	NOTCH2	20±2	17±1	−1.0	0.070
Hs.248141.0.S1_3p_at	KCNJ11	21±3	22±1	0.8	0.960
g5174532_3p_at	MC4R	7±1	5±1	−1.0	0.110
g7705548_3p_a_at	PPARG	11±1	11±1	−0.9	0.673

Data are expressed as mean ± SE (standard error of the mean) of transcript array signals of control samples and samples from type 2 diabetic subjects. Differentially expressed genes as for the lower confidence bound (LCB) (1.2) and/or the p value (p<0.05) are in bold. Ctrl: Control subjects; T2D: Type 2 diabetic subjects.

### Functional and ontologies gene sets analysis

After collapsing of the probe sets by using the gene symbols, 20,648 genes were selected; out of these genes, 9,718 (47.1%) were markers of the control phenotype and 10,930 (52.9%) were markers of the T2D phenotype. Using the phenotype-based permutation on the functional gene set analysis (C2 collection, canonical pathways, chemical and genetic perturbations), two gene sets were found enriched in samples obtained from T2D subjects and one was enriched in samples from non-diabetic controls (Table S4). In the C5 GO Biological Process collection, 13 gene sets were enriched in samples from T2D subjects and 5 were enriched in samples from controls (Table S4). In the GO Molecular Function and GO Cellular Component, 7 gene sets and 3 gene sets were respectively enriched in beta-cell samples from T2D subjects (Table S4). Changes of particular interest include three sets indicating altered JNK activity, which may have implications for susceptibility to apoptosis. Also noteworthy was the differential expression of fatty acid oxidation pathways, especially considering the findings linking altered fat metabolism with insulin secretory dysfunction [39].

### Summary

These data provide unique new information about gene expression in beta-cells of subjects with T2D. Some of the results obtained previously from preparations of isolated human islets agree with ours, but much does not, which is not surprising considering the lack of purity of these samples and the trauma to which they were exposed. Our approach also has some drawbacks, but the use of LCM on frozen sections eliminates many for the artifacts of islet isolation. Some changes were found in genes implicated in glucotoxicity, which complement existing rodent data, but the premorbid illness of the subjects may have obscured changes that might have been found with pristine specimens that might be obtained by either surgery or biopsy.

Little was found in genes associated with beta-cell replication and death, including the categories of cell cycle, apoptosis and ER stress. An important point is that there is heterogeneity in beta-cells, which is poorly understood, such that cells primed for division and death might be a small minority of the population. This fits the small number of beta-cells immunostained for CHOP (DDIT) in T2D islets [47]. Thus, it is easy to see how relevant changes of gene expression in a minority population could be missed. It is of considerable interest that upregulation of a number of metallothionein genes was found; the genes are known to be sensitive to alterations in oxidative stress.

The changes in the expression of genes associated with pancreatic regeneration were unexpected findings from the samples of these subjects with T2D. There is little to support the presence of active regeneration in T2D, but there may be a process that is somehow activated and then arrested. These findings certainly require further study.

Finally, differential gene expression was found in some of the genes linked by GWAS studies to the pathogenesis of T2D. These finding may contribute to the intense efforts currently underway to understand how these genes make their contributions to the development of T2D.

## Methods

### Ethics Statement

Tissue samples were obtained and records reviewed with IRB approval from Partners Healthcare and Joslin Diabetes Center. The study of the discarded human tissue and review of medical records was considered exempt from informed consent by both of these IRBs.

### Tissue samples

Pancreas specimens from ten non-diabetic and ten T2D subjects were studied. The pancreases from non-diabetic donors and two pancreases from T2D subjects were obtained from the New England Organ Bank and processed at the Joslin Diabetes Center. Pancreas specimens of eight T2D subjects were obtained from the University of Pisa. All the donors were White; the clinical characteristics are reported in Table 1. All the samples were processed using the same protocol. Pieces of pancreas were excised from the body of the pancreas, placed in cryomolds, embedded in Tissue-Tek OCT medium (Sakura Finetek U.S.A., Torrance, CA), immediately frozen in chilled isopentane, and stored at −80°C, pending sectioning at 8 µm [18].

### Laser Capture Microdissection (LCM)

LCM was performed using a modified protocol for human pancreatic tissue dehydration [18], [19]. Frozen pancreatic sections were fixed in 70% ethanol for 30 seconds, rinsed by 5 dips in diethylpyrocarbonate (DEPC)-treated water and dehydrated in 100% ethanol twice for 1 min, and xylene for 4 minutes, the sections were air-dried and LCM was performed using PixCell II Laser Capture Microdissection System (Arcturus Engineering, Mountain View, CA). LCM was performed by melting thermoplastic films mounted on transparent LCM caps (Arcturus) on beta–cells, identified by their intrinsic autofluorescence, in islets with no signs of amyloidal deposits. For the smallest spot size, the system was set to the following parameters: 35 mW the laser power, 2.5 msec the pulse duration and 7.5 µm the spot size. The thermoplastic film containing the microdissected cells was incubated with 10 µl of guanidine thiocyanate and polyethylene glycol octylphenol ether-based buffer (Buffer RLT, Qiagen, Valencia, CA), for 30 minutes at 42°C. Each microdissection session was performed in 15–20 min during which no more than 3 pancreatic sections were processed; each section typically contained 3–7 islets. LCM was performed on 20 pancreas sections obtained from the control donors and on 20–30 sections obtained from the diabetic subjects.

### RNA extraction, amplification biotinylation and GeneChip processing

Total RNA was extracted from each population of laser captured beta–cells using a modified protocol for RNA microisolation [76]. RNA was extracted using phenol-chloroform-isoamyl alcohol and precipitated with sodium acetate and glycogen carrier in isopropanol. After initial recovery and resuspension of the RNA pellet, genomic DNA contamination was removed by incubation with 10 units of DNase I (GenHunter, Nashville, TN) for 2 hours at 37°C, in presence of 10 units of RNase inhibitor (Life Technologies, Inc., Gaithersburg, MD). The treatment with DNase I was followed by RNA re-extraction and precipitation. The pellet was resuspended and total RNA was amplified by T7-based linear amplification using T7-oligo-dT-primers. Two rounds of amplification were performed using RiboAmp HS RNA Amplification Kits (Arcturus) [18], [19]. Amplified RNA (aRNA) quantity was evaluated spectro-photometrically by readings at 260 nm (A260) and 280 nm (A280). RNA quality was assessed by running 100 ng of aRNA on Nano LabChip of Agilent 2100 Bioanalyzer (Agilent Technologies, Inc., Santa Clara, CA). Amplified RNA (1 µg) was converted into double-stranded complementary DNA (cDNA) using the RiboAmp HS RNA Amplification Kit (Arcturus), and biotinylated complementary RNA (cRNA) was generated from cDNA by *in vitro* transcription reaction using the BioArray High Yield RNA Transcript Labeling Kit (Enzo Diagnostics, Farmingdale, NY). RNA products were purified using the MiraColTM Purification Columns (Arcturus). RNA extraction and amplification yielded aRNA with A260:A280 ratios of 2.3. The RNA integrity number (RIN) was 2.3 for most of the samples.

Biotinylated cRNA was fragmented to nucleotide stretches of 30–200 nucleotides and hybridized to the GeneChip Human X3P Array (Affymetrix, Santa Clara, CA). The Affimetrix X3P chip contains 61,359 probe sets representing 47,000 transcripts and variants, including approximately 38,500 well-characterized human genes. The probe arrays were washed and stained using the Fluidics Station 400 and scanned using the Affymetrix Gene Chip Scanner 3000 (Gene Chip Expression Analysis *Technical Manual*, Affymetrix). Microarray experiments of four samples from T2D subjects and four samples from non-diabetic controls were run at the Genomics Core facility of the Massachusetts General Hospital, the remaining microarrays, six samples from diabetic subjects and six from non-diabetic controls were run at the Genomics Core of the Joslin Diabetes Center. Principal component analysis was performed by the Rosetta Resolver System (Rosetta Biosoftware, Microsoft, Seattle, WA) to assess the variation in the expression of genes among the different samples.

### Microarray data analysis

Array data were normalized and comparisons were performed using the DNA-Chip Analyzer (dChip) software (Harvard School of Public Health, Boston, MA). dChip software implements invariant set normalization and probe-level model-based expression analysis on multiple arrays, and computes the t-statistic and the p-value based on the t-distribution. Computation of standard errors for expression indexes allows calculating confidence intervals for fold changes [77], [78]. Lower confidence bound (LCB) and p-value were used to assess differentially expressed genes; cutoffs of 1.2 for LCB and p<0.05 are often used. Hierarchical clustering was performed using dChip software. Principal component analysis was performed by the Rosetta Resolver System (Rosetta Biosoftware, Microsoft, Seattle, WA) to assess the variation in the expression of genes among the different samples. Changes in the expression of functionally related genes at the genome-wide expression profile level were detected using Gene Set Enrichment Analysis (GSEA, versions 2 and 2.5, Broad Institute, Cambridge, MA). For the analysis, genes represented by more than one probe were collapsed to the probe with the maximum value using the gene symbols [79]. Genes were ranked based on values estimated by Affymetrix Micro Array Suite (MAS) 5.0 and scaled to the value of 1500. Functional (C2 collection: 1456 gene sets) and gene ontology (GO) (C5 collection: 825 Biological Process Ontology gene sets, BP; 396 Molecular Function Ontology gene sets, MF; 233 Cellular Components Ontology gene sets, CC) gene sets, restricted to a minimum of 15 and a maximum of 1000 gene set size, were analyzed. A nominal p-value<0.01 at multiple hypothesis testing false discovery rate (FDR)≤25% was used as parameter to detect differentially regulated gene sets.

### Quantitative analysis of selected transcripts by real-time PCR

PCR measurements of selected transcripts using amplified RNA were determined on samples from four T2D subjects and four non-diabetic controls. cDNA templates were synthesized from 1 µg of aRNA using TaqMan Reverse Transcription Reagents (Applied Biosystems). TaqMan Universal PCR Master Mix (Applied Biosystems) was used to perform real-time PCR in the presence of 0.2 ng cDNA, 1 µM primers and 0.25 µM probe in a total volume of 20 µl. The housekeeping gene ribosomal protein L32 (RPL32) transcript was used as a reference. Primers and probes were designed using Primer Express software (Applied Biosystems) and were purchased from MWG-Biotech Inc. (MWG-Biotech Inc., High Point, NC). Primers and probe sequences are reported in Table 3. Quantitative analysis of gene expression was performed using the Applied Biosystems 7300 Real Time PCR System (Applied Biosystems). For each sample, triplicate amplifications were performed and average measurements were taken for data analysis. The N-fold differential expression was assessed by 2^−ΔΔCT^ method and the statistical significance was evaluated by the two-tailed Student's t-test using the dCt values.

**Table 3:** Primers and probes used for real-time PCR.

Transcript	GeneBank no.	Primer	Primer sequence (5′-3′)	Probe sequence (5′-3′)
REG1A	NM_002909	F	CCTCCATGACCCCAAAAAGA	CGCCGCTGGCACTGGAGCA
		R	AATGCCCCAGGACTTGTAGGA	
REG1B	NM_006507	F	GGTCCTGCAATTACTATGAAGTCAAA	CTCCAACTCAGTTCAGACCATCTCCTCCC
		R	AAGATCAGCGATGCAAACTCATT	
REG3A	NM_002580	F	GGTTACCCTATGTCTGCAAGTTCA	TGACTAGTGCAGGAGGGAAGTCAGCAGC
		R	GATGAGTTGCACACCAAACACA	
REG3G	NM_001008387	F	CCCCACACAGGGCTCTGA	CCTGATGGAGATGGATGGGAGTGGAG
		R	CCATGCAAAGTAATTCATCACATCA	
SOX9	NM_000346	F	CCACTGATTGGCCACAAGTG	AATGCGCTTGGATAGGTCATGTTTGTGTC
		R	GCAACTCGTACCCAAATTTCCA	
MMP7	NM_002423	F	TGTATGCTGCAACTCATGAACTTG	CCATTCTTTGGGTATGGGACATTCCTCTG
		R	AGGTTGGATACATCACTGCATTAGG	
RPL32	NM_000994	F	CTGGCCATCAGAGTCACCAA	CCCAATGCCAGGCTGCGCA
		R	TGAGCTGCCTACTCATTTTCTTCA	

Primers and probes were designed using Primer Express software (Applied Biosystems); the oligonucleotide sequences for forward (F) and reverse (R) primers and the probe were designed within 20–200 bases close to the 3′ end of the transcript with one of the primers or the probe spanning two adjacent exons.

### Data analysis

Data are expressed as mean (M) ± standard deviation (SD) or standard error of the mean (SE), as indicated. The two-tailed Student's t-test was used to compare data from pancreas donors.

## Supporting Information

Table S1 Gene expression of molecules involved in glucose and lipid metabolism, and beta-cell channels structure and function, and of the adrenergic receptor, insulin signaling molecules, ARNT, ARNT2 and HIF1A.
(0.09 MB DOC)

Table S2 Gene expression of hormones, receptors, transcription factors, and molecules involved in exocytosis, cell cycle, WNT and Notch signaling, TGF-beta signaling and tumor suppression.
(0.15 MB DOC)

Table S3 Gene expression of molecules involved in ER stress, apoptosis, oxidative stress, islet development and regeneration.
(0.11 MB DOC)

Table S4 GSEA results with FDR≤0.25.
(0.08 MB DOC)
