# Supplementary material for: Gene Expression Profiles of Beta-Cell Enriched Tissue Obtained by Laser Capture Microdissection from Subjects with Type 2 Diabetes
Source: PLoS One. 2010 Jul 13;5(7):e11499. doi: 10.1371/journal.pone.0011499 (PMC2903480; doi:10.1371/journal.pone.0011499)
Supplement: Table S1 — Gene expression of molecules involved in glucose and lipid metabolism, and beta-cell channels structure and function, and of the adrenergic receptor, insulin signaling molecules, ARNT, ARNT2 and HIF1A. (0.09 MB DOC) [file pone.0011499.s001.doc]

**Table S1. Gene expression of molecules involved in glucose and lipid metabolism, and beta-cell channels structure and function, and of the adrenergic receptor, insulin signaling molecules, ARNT, ARNT2 and HIF1A**

| Probe ID | Gene symbol | Ctrl | T2D | LCB | p value |
| --- | --- | --- | --- | --- | --- |
| **Glucose metabolism** | | | | | |
| g5730050_3p_a_at | GLUT1 | 163 ± 6 | 153 ± 6 | -1.0 | 0.271 |
| **g4557850_3p_at** | **GLUT2** | **320 ± 41** | **94 ± 19** | **-2.4** | **0.000** |
| g183226_3p_a_at | GCK | 184 ± 16 | 202 ± 19 | 0.9 | 0.473 |
| g4504086_3p_s_at | GPI | 453 ± 50 | 487 ± 67 | 0.8 | 0.687 |
| g975289_3p_a_at | PFKM | 1657 ± 88 | 1611 ± 78 | -0.9 | 0.699 |
| g11321600_3p_at | PFKP | 464 ± 43 | 527 ± 45 | 0.9 | 0.323 |
| g4505746_3p_s_at | PFKL | 439 ± 49 | 506 ± 32 | 0.9 | 0.270 |
| **g4557306_3p_a_at** | **ALDOB** | **2106 ± 451** | **3631 ± 511** | **1.2** | **0.038** |
| g4505764_3p_a_at | PGM1 | 2492 ± 130 | 2436 ± 109 | -0.9 | 0.746 |
| **201231_3p_s_at** | **ENO1** | **77 ± 6** | **57 ± 5** | **-1.1** | **0.020** |
| **g5031856_3p_a_at** | **LDHA** | **426 ± 34** | **721 ± 123** | **1.2** | **0.042** |
| Hs.148438.0.A1_3p_at | PC | 26 ± 1 | 27 ± 1 | 0.9 | 0.718 |
| **g4505638_3p_a_at** | **PCK1** | **178 ± 50** | **662 ± 101** | **2.3** | **0.001** |
| 1559861_3p_at | FBP2 | 23 ± 1 | 24 ± 1 | 1.0 | 0.309 |
| 1555612_3p_s_at | G6PC | 21 ± 1 | 19 ± 1 | -1.0 | 0.225 |
| Hs.294005.0.A1_3p_s_at | G6PC3 | 41 ± 4 | 33 ± 2 | -1.0 | 0.109 |
| **g181574_3p_at** | **DLD** | **865 ± 49** | **694 ± 38** | **-1.1** | **0.013** |
| **g4501866_3p_at** | **ACO2** | **94 ± 4** | **81 ± 5** | **-1.0** | **0.048** |
| **g2588778_3p_a_at** | **SDHC** | **362 ± 18** | **435 ± 20** | **1.1** | **0.015** |
| g5174470_3p_at | IDH1 | 1346 ± 35 | 1460 ± 80 | 1.0 | 0.218 |
| Hs.111076.1.S1_3p_at | MDH2 | 837 ± 100 | 822 ± 110 | -0.8 | 0.920 |
| **g1020314_3p_a_at** | **GPD2** | **72 ± 5** | **52 ± 5** | **-1.1** | **0.011** |
| **g13435400_3p_a_at** | **ME1** | **337 ± 44** | **219 ± 23** | **-1.2** | **0.035** |
| **g4501864_3p_s_at** | **ACLY** | **3052 ± 133** | **2434 ± 107** | **-1.1** | **0.002** |
| g4557816_3p_at | OXCT1 | 291 ± 26 | 246 ± 30 | -0.9 | 0.270 |
| g4502852_3p_a_at | CKM | 20 ± 1 | 23 ± 2 | 0.9 | 0.329 |
| g4502850_3p_at | CKB | 1549 ± 231 | 1125 ± 156 | -1.0 | 0.148 |
| **Lipid metabolism** | | | | | |
| **Hs.11050.2.A2_3p_s_at** | **FASN** | **62 ± 11** | **136 ± 21** | **1.5** | **0.007** |
| **g2078328_3p_a_at** | **HADH** | **685 ± 44** | **430 ± 27** | **-1.4** | **0.000** |
| **g7415720_3p_a_at** | **SCD** | **2827 ± 280** | **1847 ± 150** | **-1.2** | **0.008** |
| Hs.63788.0.S1_3p_a_at | PCCB | 374 ± 17 | 374 ± 15 | -0.9 | 0.996 |
| **Channels and Adrenergic receptor** | | | | | |
| **g3643189_3p_at** | **SUR1** | **4030 ± 177** | **3098 ± 104** | **-1.2** | **0.000** |
| **Hs.23838.1.A2_3p_at** | **CACNA1D** | **175 ± 13** | **118 ± 12** | **-1.2** | **0.005** |
| **g4504794_3p_a_at** | **ITPR3** | **1338 ± 65** | **1077 ± 64** | **-1.2** | **0.010** |
| **g4557688_3p_a_at** | **KCNQ1** | **138 ± 25** | **466 ± 82** | **2.2** | **0.003** |
| **g13447750_3p_at** | **ADRA2A** | **295 ± 24** | **210 ± 16** | **-1.2** | **0.009** |
| **Insulin signaling** | | | | | |
| **Hs.98401.0.S1_3p_at** | **INSR** | **212 ± 21** | **272 ± 18** | **1.1** | **0.043** |
| **Hs.239176.0.S3_3p_at** | **IGF1R** | **104 ± 16** | **189 ± 22** | **1.3** | **0.007** |
| **g5031798_3p_a_at** | **INPP5D** | **126 ± 12** | **242 ± 34** | **1.4** | **0.006** |
| **g4755141_3p_a_at** | **INPPL1** | **98 ± 8** | **124 ± 6** | **1.1** | **0.017** |
| **Hs.37003.0.S1_3p_at** | **HRAS** | **39 ± 4** | **53 ± 5** | **1.1** | **0.039** |
| **g4506400_3p_a_at** | **RAF1** | **321 ± 26** | **393 ± 18** | **1.1** | **0.035** |
| **g9257221_3p_a_at** | **FOXO1** | **1737 ± 97** | **1384 ± 101** | **-1.1** | **0.021** |
| g4885060_3p_a_at | AKT1 | 592 ± 81 | 538 ± 29 | -0.8 | 0.548 |
| Hs.182278.4.S1_3p_a_at | AKT2 | 892 ± 79 | 1085 ± 92 | 1.0 | 0.129 |
| Hs.300642.0.S1_3p_at | AKT3 | 290 ± 27 | 255 ± 18 | -0.9 | 0.298 |
| **ARNT ARNT 2 and HNF-1alpha** | | | | | |
| Hs.166172.2.S1_3p_at | ARNT | 56 ± 4 | 60 ± 5 | 0.9 | 0.523 |
| g7662049_3p_s_at | ARNT2 | 954 ± 60 | 873 ± 42 | -1.0 | 0.284 |
| g4504384_3p_at | HIF1A | 2248 ± 61 | 2201 ± 96 | -0.9 | 0.683 |

Data are expressed as mean ± SE (standard error of the mean) of transcript array signals of control samples and samples from type 2 diabetic subjects. Differentially expressed genes as for the lower confidence bound (LCB) (1.2) and/or the p value (p < 0.05) are in bold. Ctrl: Control subjects; T2D: Type 2 diabetic subjects; LCB: Lower Confidence Bound.
