# Supplementary material for: Gene Expression Profiles of Beta-Cell Enriched Tissue Obtained by Laser Capture Microdissection from Subjects with Type 2 Diabetes
Source: PLoS One. 2010 Jul 13;5(7):e11499. doi: 10.1371/journal.pone.0011499 (PMC2903480; doi:10.1371/journal.pone.0011499)
Supplement: Table S2 — Gene expression of hormones, receptors, transcription factors, and molecules involved in exocytosis, cell cycle, WNT and Notch signaling, TGF-beta signaling and tumor suppression. (0.15 MB DOC) [file pone.0011499.s002.doc]

**Table S2. Gene expression of hormones, receptors, transcription factors, and molecules involved in exocytosis, cell cycle, WNT and Notch signaling, TGF-beta signaling and tumor suppression.**

| Probe ID | Gene symbol | Ctrl | T2D | LCB | p value |
| --- | --- | --- | --- | --- | --- |
| **Islet hormones and receptors** | | | | | |
| g4557670_3p_at | INS | 5182 ± 246 | 5570 ± 373 | 0.9 | 0.398 |
| **g4557654_3p_at** | **IAPP** | **8919 ± 586** | **6358 ± 768** | **-1.1** | **0.017** |
| g4503944_3p_at | GCG | 9792 ± 53 | 9652 ± 61 | -1.0 | 0.101 |
| Hs.12409.0.S1_3p_at | SST | 5231 ± 66 | 5559 ± 177 | 1.0 | 0.110 |
| g190301_3p_at | PPY | 48 ± 6 | 49 ± 5 | 0.8 | 0.977 |
| g439689_3p_at | GCGR | 211 ± 8 | 207 ± 9 | -0.9 | 0.721 |
| g405081_3p_a_at | GLP1R | 57 ± 2 | 63 ± 4 | 1.0 | 0.208 |
| g4503998_3p_at | GIPR | 5 ± 0 | 4 ± 1 | -0.9 | 0.239 |
| **Islet transcription factors** | | | | | |
| **g7019436_3p_at** | **PDX1** | **27 ± 3** | **42 ± 3** | **1.3** | **0.002** |
| g4505400_3p_at | NKX2-2 | 787 ± 85 | 590 ± 56 | -1.1 | 0.072 |
| g5453787_3p_at | NKX6-1 | 9 ± 1 | 10 ± 2 | 0.7 | 0.854 |
| g4505376_3p_at | NEUROD1 | 1923 ± 102 | 1846 ± 123 | -0.9 | 0.640 |
| g5453849_3p_at | PAX4 | 17 ± 3 | 22 ± 3 | 0.9 | 0.245 |
| **g4505614_3p_s_at** | **PAX6** | **2384 ± 149** | **3302 ± 239** | **1.2** | **0.005** |
| **g184264_3p_at** | **HNF1A** | **104 ± 4** | **82 ± 5** | **-1.1** | **0.002** |
| Hs.54424.3.S1_3p_a_at | HNF4A | 43 ± 2 | 44 ± 3 | 0.9 | 0.755 |
| g10337610_3p_at | NEUROG3 | 7 ± 1 | 7 ± 1 | -0.7 | 0.696 |
| **Hs.103538.0.S1_3p_at** | **ARX** | **634 ± 40** | **833 ± 65** | **1.1** | **0.019** |
| g4885446_3p_a_at | MAFB | 2475 ± 194 | 2280 ± 199 | -0.9 | 0.491 |
| **Exocytosis** | | | | | |
| g4759181_3p_a_at | STX1A | 2358 ± 236 | 1974 ± 162 | -1.0 | 0.199 |
| **g4507098_3p_a_at** | **SNAP25** | **3382 ± 277** | **2678 ± 144** | **-1.1** | **0.041** |
| g4507296_3p_a_at | STXBP1 | 2762 ± 100 | 2698 ± 77 | -1.0 | 0.618 |
| **Hs.23179.0.A1_3p_a_at** | **SYT5** | **23 ± 1** | **30 ± 2** | **1.1** | **0.006** |
| Hs.75667.0.S1_3p_at | SYP | 587 ± 102 | 560 ± 49 | -0.7 | 0.811 |
| Hs.20021.1.S1_3p_at | VAMP1 | 125 ± 4 | 138 ± 7 | 1.0 | 0.145 |
| Hs.194534.1.A1_3p_a_at | VAMP2 | 556 ± 43 | 517 ± 23 | -0.9 | 0.430 |
| g13097737_3p_at | VAMP3 | 1454 ± 54 | 1430 ± 49 | -0.9 | 0.742 |
| **g5454147_3p_at** | **UNC13B** | **523 ± 29** | **348 ± 19** | **-1.3** | **0.000** |
| Hs.8059.0.S2_3p_at | SYT4 | 2694 ± 70 | 2753 ± 85 | 1.0 | 0.596 |
| g11079227_3p_at | NSF | 1598 ± 69 | 1430 ± 40 | -1.0 | 0.053 |
| g12654654_3p_a_at | NAPA | 1088 ± 75 | 1154 ± 62 | 0.9 | 0.510 |
| **Cell cycle genes** | | | | | |
| g4502616_3p_a_at | CCND2 | 1706 ± 82 | 1792 ± 92 | 0.9 | 0.492 |
| g4502618_3p_at | CCND3 | 2079 ± 176 | 2213 ± 219 | 0.9 | 0.640 |
| g4502734_3p_s_at | CDK4 | 218 ± 12 | 238 ± 27 | 0.9 | 0.522 |
| Hs.180059.0.S3_3p_at | CDK6 | 346 ± 37 | 351 ± 25 | 0.8 | 0.907 |
| **g180177_3p_at** | **CDK2** | **24 ± 2** | **31 ± 2** | **1.1** | **0.018** |
| g4502748_3p_at | p16 (INK4a) | 317 ± 32 | 426 ± 44 | 1.1 | 0.060 |
| **g11386202_3p_a_at** | **p21 (Cip1)** | **2011 ± 232** | **2622 ± 122** | **1.1** | **0.035** |
| g4506434_3p_at | RB1 | 241 ± 16 | 241 ± 25 | 0.8 | 0.990 |
| **Hs2.87.2.S1_3p_s_at** | **RBL1** | **42 ± 3** | **32 ± 3** | **-1.1** | **0.026** |
| Hs.79362.0.S3_3p_at | RBL2 | 1283 ± 60 | 1309 ± 55 | 0.9 | 0.746 |
| **g7705484_3p_a_at** | **CDK5RAP1** | **371 ± 22** | **524 ± 38** | **1.2** | **0.003** |
| **Hs.32360.2.S1_3p_a_at** | **CDK5RAP2** | **626 ± 44** | **818 ± 38** | **1.1** | **0.004** |
| **g13376787_3p_a_at** | **CDK5RAP3** | **1141 ± 66** | **1400 ± 68** | **1.1** | **0.014** |
| g4758323_3p_a_at | EZH2 | 36 ± 3 | 40 ± 3 | 0.9 | 0.299 |
| Hs2.407338.1.S1_3p_s_at | MLL | 24 ± 2 | 25 ± 2 | 0.9 | 0.797 |
| g4506038_3p_a_at | PRC1 | 112 ± 4 | 101 ± 6 | -1.0 | 0.165 |
| Hs.103915.0.S1_3p_at | JMJD3 | 38 ± 2 | 37 ± 2 | -0.9 | 0.863 |
| g4885094_3p_at | BMI1 | 2368 ± 52 | 2247 ± 96 | -1.0 | 0.286 |
| **WNT signaling pathway** | | | | | |
| g4885654_3p_at | WNT1 | 17 ± 1 | 17 ± 2 | 0.8 | 0.878 |
| g4507926_3p_at | WNT2 | 52 ± 2 | 55 ± 4 | 0.9 | 0.547 |
| **4809630C_3p_s_at** | **WNT6** | **60 ± 4** | **22 ± 4** | **-2.1** | **0.000** |
| **4851107C_3p_at** | **DVL2** | **517 ± 30** | **650 ± 49** | **1.1** | **0.035** |
| **Hs.278503.1.A1_3p_s_at** | **DKK3** | **807 ± 138** | **2283 ± 340** | **1.9** | **0.002** |
| **g7657024_3p_at** | **DKK4** | **123 ± 3** | **134 ± 3** | **1.0** | **0.010** |
| **Hs.70823.0.S3_3p_at** | **SULF1** | **75 ± 17** | **135 ± 13** | **1.3** | **0.012** |
| **g6912383_3p_at** | **FZD4** | **254 ± 14** | **174 ± 18** | **-1.2** | **0.003** |
| **Hs.31386.0.S1_3p_a_at** | **SFRP2** | **104 ± 4** | **135 ± 9** | **1.1** | **0.008** |
| **g8400734_3p_a_at** | **SFRP5** | **51 ± 7** | **111 ± 20** | **1.5** | **0.015** |
| Hs.173638.0.S3_3p_at | TCF7L2 | 1111 ± 72 | 1112 ± 77 | 0.9 | 0.995 |
| 221021_3p_s_at | CTNNBL1 | 132 ± 10 | 138 ± 11 | 0.9 | 0.690 |
| **Notch signaling pathway** | | | | | |
| g8923012_3p_at | NOTCH1 | 95 ± 4 | 96 ± 12 | 0.8 | 0.937 |
| g11275977_3p_a_at | NOTCH2 | 20 ± 2 | 17 ± 1 | -1.0 | 0.070 |
| g4557798_3p_a_at | NOTCH3 | 203 ± 20 | 242 ± 30 | 0.9 | 0.291 |
| **Hs.323231.0.S1_3p_at** | **NOTCH2NL** | **241 ± 29** | **436 ± 57** | **1.3** | **0.009** |
| g6180181_3p_a_at | DLL1 | 120 ± 17 | 131 ± 17 | 0.8 | 0.663 |
| Hs.127792.0.A1_3p_a_at | DLL3 | 40 ± 2 | 44 ± 2 | 1.0 | 0.214 |
| g10567109_3p_at | DLL4 | 63 ± 2 | 67 ± 3 | 1.0 | 0.241 |
| g562105_3p_a_at | DLK1 | 8724 ± 291 | 8600 ± 385 | -0.9 | 0.800 |
| g1695273_3p_a_at | JAG1 | 56 ± 2 | 61 ± 6 | 0.9 | 0.439 |
| **g2605944_3p_s_at** | **JAG2** | **110 ± 3** | **127 ± 5** | **1.1** | **0.015** |
| g8400709_3p_a_at | HES1 | 79 ± 9 | 121 ± 23 | 1.0 | 0.115 |
| g7549812_3p_a_at | PSEN1 | 355 ± 16 | 418 ± 39 | 1.0 | 0.164 |
| g1079575_3p_s_at | PSEN2 | 1694 ± 60 | 1657 ± 88 | -0.9 | 0.732 |
| g4505158_3p_a_at | MFNG | 15 ± 1 | 16 ± 2 | 0.8 | 0.679 |
| **TGF-beta signaling** | | | | | |
| **Hs.211578.0.S3_3p_at** | **SMAD3** | **104 ± 3** | **88 ± 3** | **-1.1** | **0.001** |
| **Hs.165210.0.A1_3p_at** | **SMAD5** | **83 ± 8** | **54 ± 4** | **-1.3** | **0.006** |
| g5902814_3p_a_at | BMP1 | 19 ± 1 | 18 ± 1 | -0.8 | 0.990 |
| **g5902809_3p_at** | **BMP1** | **63 ± 3** | **84 ± 5** | **1.2** | **0.001** |
| g10835090_3p_a_at | BMP5 | 429 ± 27 | 715 ± 129 | 1.2 | 0.056 |
| **g4758051_3p_at** | **CRABP1** | **36 ± 2** | **27 ± 2** | **-1.1** | **0.005** |
| **g4759283_3p_s_at** | **UCHL1** | **4215 ± 57** | **3903 ± 104** | **-1.0** | **0.020** |
| **g5174740_3p_at** | **UCHL3** | **865 ± 40** | **745 ± 37** | **-1.0** | **0.041** |
| **g6912533_3p_at** | **BAMBI** | **169 ± 22** | **274 ± 36** | **1.2** | **0.025** |
| **g13477368_3p_at** | **INHBE** | **14 ± 5** | **48 ± 11** | **1.8** | **0.014** |
| **Hs.92614.1.S1_3p_at** | **LASS1** | **231 ± 28** | **377 ± 33** | **1.3** | **0.003** |
| **g4557544_3p_a_at** | **EDN3** | **3999 ± 291** | **1914 ± 275** | **-1.6** | **0.000** |
| g4557546_3p_at | EDNRB | 64 ± 7 | 55 ± 6 | -0.9 | 0.342 |
| **Hs.37054.0.S2_3p_at** | **EFNA3** | **93 ± 11** | **181 ± 20** | **1.5** | **0.002** |
| **Tumor suppressor genes** | | | | | |
| g4506434_3p_at | RB1 | 241 ± 16 | 241 ± 25 | 0.8 | 0.990 |
| g8400737_3p_at | TP53 | 34 ± 6 | 41 ± 5 | 0.9 | 0.341 |
| g4506248_3p_at | PTEN | 136 ± 12 | 123 ± 10 | -0.9 | 0.441 |
| Hs.75081.0.S2_3p_a_at | APC | 137 ± 15 | 116 ± 10 | -0.9 | 0.248 |
| g7019352_3p_at | MED23 | 295 ± 26 | 318 ± 29 | 0.9 | 0.556 |
| g4758795_3p_at | DRG1 | 1240 ± 49 | 1190 ± 44 | -1.0 | 0.459 |
| g4757943_3p_at | CD81 | 7791 ± 174 | 7549 ± 141 | -1.0 | 0.293 |
| g4557796_3p_at | NME1 | 297 ± 24 | 365 ± 54 | 0.9 | 0.279 |
| g4505408_3p_s_at | NME2 | 2614 ± 107 | 2806 ± 171 | 1.0 | 0.358 |
| Hs.6441.0.A1_3p_a_at | TIMP2 | 586 ± 35 | 633 ± 41 | 0.9 | 0.391 |
| g6552300_3p_a_at | BRCA1 | 228 ± 40 | 219 ± 49 | -0.7 | 0.881 |

Data are expressed as mean ± SE (standard error of the mean) of transcript array signals of control samples and samples from type 2 diabetic subjects. Differentially expressed genes as for the lower confidence bound (LCB) (1.2) and/or the p value (p < 0.05) are in bold. Ctrl: Control subjects; T2D: Type 2 diabetic subjects.
