# Supplementary material for: Gene Expression Profiles of Beta-Cell Enriched Tissue Obtained by Laser Capture Microdissection from Subjects with Type 2 Diabetes
Source: PLoS One. 2010 Jul 13;5(7):e11499. doi: 10.1371/journal.pone.0011499 (PMC2903480; doi:10.1371/journal.pone.0011499)
Supplement: Table S3 — Gene expression of molecules involved in ER stress, apoptosis, oxidative stress, islet development and regeneration. (0.11 MB DOC) [file pone.0011499.s003.doc]

**Table S3. Gene expression of molecules involved in ER stress, apoptosis, oxidative stress, islet development and regeneration.**

| Probe ID | Gene symbol | Ctrl | T2D | LCB | p value |
| --- | --- | --- | --- | --- | --- |
| **ER stress genes** | | | | | |
| **g7706436_3p_s_at** | **MAN1B1** | **215 ± 15** | **341 ± 31** | **1.3** | **0.003** |
| 214703_3p_s_at | **MAN2B2** | **193 ± 12** | **251 ± 18** | **1.1** | **0.017** |
| **g13376781_3p_s_at** | **EDEM3** | **85 ± 6** | **62 ± 5** | **-1.1** | **0.011** |
| **Hs.188228.0.S1_3p_at** | **ERO1LB** | **4538 ± 257** | **3702 ± 243** | **-1.1** | **0.030** |
| **Hs.293902.0.A1_3p_at** | **ERP27** | **74 ± 15** | **217 ± 61** | **1.5** | **0.046** |
| **g5453979_3p_a_at** | **DNAJC3** | **57 ± 4** | **69 ± 4** | **1.1** | **0.033** |
| g4827057_3p_s_at | XBP1 | 4867 ± 145 | 4904 ± 141 | 0.9 | 0.858 |
| g6671584_3p_s_at | ATF6 | 152 ± 9 | 154 ± 14 | 0.8 | 0.903 |
| g13182760_3p_at | EIF2A | 364 ± 31 | 316 ± 28 | -0.9 | 0.265 |
| g11386200_3p_at | UGCGL2 | 217 ± 16 | 165 ± 22 | -1.0 | 0.079 |
| g13177717_3p_at | DDIT3 | 98 ± 6 | 100 ± 9 | 0.9 | 0.834 |
| Hs.75410.4.A1_3p_at | HSPA5 | 105 ± 13 | 85 ± 10 | -0.9 | 0.242 |
| Hs.82689.0.S1_3p_a_at | HSP90B1 | 352 ± 45 | 302 ± 31 | -0.9 | 0.379 |
| Hs.267445.0.S2_3p_at | MAPK8 | 141 ± 10 | 149 ± 16 | 0.9 | 0.675 |
| **g12653312_3p_at** | **PDIA4** | **413 ± 53** | **644 ± 66** | **1.2** | **0.014** |
| g13376995_3p_at | WFS1 | 981 ± 75 | 1145 ± 90 | 1.0 | 0.178 |
| **Apoptosis** | | | | | |
| g4557354_3p_at | BCL2 | 56 ± 9 | 67 ± 9 | 0.8 | 0.420 |
| Hs.305890.1.S1_3p_at | BCL2L1  (BCLX) | 24 ± 3 | 25 ± 8 | 0.5 | 0.929 |
| g1683636_3p_s_at | BAD | 100 ± 6 | 116 ± 7 | 1.0 | 0.114 |
| g4502362_3p_at | BAK1 | 11 ± 1 | 13 ± 2 | 0.9 | 0.452 |
| g841237_3p_a_at | BAX | 20 ± 1 | 21 ± 2 | 0.9 | 0.709 |
| g1944417_3p_at | BCL2L2 | 431 ± 20 | 431 ± 19 | -0.9 | 0.999 |
| **g10092609_3p_at** | **BOK** | **182 ± 7** | **162 ± 4** | **-1.0** | **0.036** |
| **g4507582_3p_a_at** | **FAS** | **3 ± 1** | **10 ± 2** | **1.7** | **0.003** |
| Hs.82359.0.S1_3p_a_at | FAS | 22 ± 4 | 22 ± 3 | 0.7 | 0.965 |
| Hs2.202657.5.S1_3p_s_at | BIM | 142 ± 11 | 210 ± 31 | 1.1 | 0.063 |
| Hs.84063.0.S1_3p_at | BIM | 219 ± 22 | 241 ± 21 | 0.9 | 0.478 |
| g4557360_3p_at | BID | 39 ± 2 | 42 ± 3 | 0.9 | 0.493 |
| g4757911_3p_at | CASP3 | 64 ± 9 | 69 ± 7 | 0.8 | 0.692 |
| g12653078_3p_a_at | CASP6 | 132 ± 10 | 120 ± 12 | -0.9 | 0.432 |
| g4502580_3p_a_at | CASP7 | 160 ± 25 | 195 ± 13 | 0.9 | 0.243 |
| g1145290_3p_a_at | BIRC3 | 87 ± 10 | 116 ± 15 | 1.0 | 0.122 |
| g10442005_3p_s_at | BCL2A1 | 502 ± 18 | 521 ± 43 | 0.9 | 0.694 |
| g5454131_3p_a_at | TNFAIP3 | 310 ± 59 | 299 ± 35 | -0.7 | 0.872 |
| g2429153_3p_at | CFLAR | 727 ± 86 | 924 ± 67 | 1.0 | 0.087 |
| **g7416052_3p_a_at** | **BIRC5** | **78 ± 4** | **90 ± 3** | **1.0** | **0.033** |
| **g4507678_3p_a_at** | **TRAF3** | **25 ± 1** | **36 ± 3** | **1.2** | **0.003** |
| **g13378136_3p_s_at** | **TRADD** | **222 ± 14** | **297 ± 19** | **1.2** | **0.005** |
| **Hs.181077.0.S1_3p_a_at** | **TRAF7** | **203 ± 14** | **294 ± 32** | **1.2** | **0.025** |
| **Hs.7446.1.S1_3p_a_at** | **TRAF3IP2** | **252 ± 24** | **153 ± 10** | **-1.4** | **0.002** |
| **Oxidative stress** | | | | | |
| **g11345419_3p_at** | **TXN** | **912 ± 39** | **776 ± 28** | **-1.1** | **0.011** |
| **g9280552_3p_a_at** | **TXN2** | **74 ± 2** | **85 ± 3** | **1.1** | **0.009** |
| Hs.211929.0.S2_3p_at | TXN2 | 456 ± 31 | 501 ± 41 | 0.9 | 0.394 |
| g5454161_3p_a_at | TXNIP | 1555 ± 122 | 1426 ± 185 | -0.9 | 0.569 |
| g4507746_3p_at | TXNRD1 | 1259 ± 35 | 1139 ± 55 | -1.0 | 0.084 |
| g6006000_3p_a_at | GPX3 | 113 ± 5 | 138 ± 17 | 1.0 | 0.196 |
| g4504106_3p_at | GPX4 | 268 ± 13 | 274 ± 41 | 0.8 | 0.896 |
| g4557013_3p_at | CAT | 600 ± 40 | 578 ± 27 | -0.9 | 0.661 |
| g4504436_3p_at | HMOX1 | 9 ± 1 | 12 ± 2 | 1.1 | 0.072 |
| **Hs.318885.1.S1_3p_a_at** | **SOD2** | **134 ± 16** | **210 ± 24** | **1.2** | **0.019** |
| Hs.318885.2.S1_3p_a_at | SOD2 | 107 ± 14 | 133 ± 27 | 0.8 | 0.401 |
| **g4507150_3p_at** | **SOD3** | **202 ± 25** | **281 ± 27** | **1.1** | **0.044** |
| **Hs.74170.0.S1_3p_x_at** | **MT1E** | **1283 ± 299** | **2287 ± 361** | **1.2** | **0.047** |
| g10835229_3p_a_at | MT1G | 259 ± 79 | 498 ± 89 | 1.1 | 0.059 |
| **Hs.188518.0.S1_3p_at** | **MT1M** | **95 ± 19** | **190 ± 37** | **1.2** | **0.039** |
| **g4505270_3p_at** | **MT1X** | **1161 ± 261** | **2132 ± 291** | **1.2** | **0.023** |
| **Hs.118786.0.S1_3p_at** | **MT2A** | **2591 ± 541** | **4983 ± 651** | **1.3** | **0.011** |
| g10835084_3p_x_at | MT1H | 67 ± 5 | 93 ± 12 | 1.1 | 0.077 |
| **g13310411_3p_at** | **MT1P2** | **316 ± 59** | **656 ± 115** | **1.4** | **0.020** |
| **Hs.21326.0.A1_3p_at** | **PPM1E** | **870 ± 73** | **516 ± 42** | **-1.4** | **0.001** |
| g9790904_3p_at | GADD45A | 536 ± 55 | 679 ± 53 | 1.0 | 0.077 |
| **g12061050_3p_a_at** | **GADD45B** | **485 ± 46** | **717 ± 60** | **1.2** | **0.007** |
| **g9790905_3p_at** | **GADD45G** | **124 ± 13** | **238 ± 42** | **1.3** | **0.025** |
| **Genes involved in pancreatic islet development and regeneration** | | | | | |
| **g5764554_3p_at** | **REG1A** | **620 ± 153** | **2601 ± 479** | **2.6** | **0.002** |
| **g10835247_3p_at** | **REG1B** | **2036 ± 579** | **4859 ± 398** | **1.6** | **0.001** |
| **g4505604_3p_at** | **REG3A** | **563 ± 423** | **2147 ± 463** | **1.5** | **0.021** |
| **Hs.312684.0.A1_3p_at** | **REG3G** | **73 ± 41** | **325 ± 103** | **1.7** | **0.043** |
| **Hs.2316.0.S1_3p_a_at** | **SOX9** | **72 ± 17** | **171 ± 23** | **1.6** | **0.003** |
| **g13027804_3p_at** | **MMP7** | **25 ± 14** | **185 ± 46** | **3.2** | **0.007** |

Data are expressed as mean ± SE (standard error of the mean) of transcript array signals of control samples and samples from type 2 diabetic subjects. Differentially expressed genes as for the lower confidence bound (LCB) (1.2) and/or the p value (p < 0.05) are in bold. Ctrl: Control subjects; T2D: Type 2 diabetic subjects.
