# Supplementary material for: Gene Expression Profiles of Beta-Cell Enriched Tissue Obtained by Laser Capture Microdissection from Subjects with Type 2 Diabetes
Source: PLoS One. 2010 Jul 13;5(7):e11499. doi: 10.1371/journal.pone.0011499 (PMC2903480; doi:10.1371/journal.pone.0011499)
Supplement: Table S4 — GSEA results with FDR≤0.25. (0.08 MB DOC) [file pone.0011499.s004.doc]

**Table S4**. GSEA results with FDR ≤ 0.25.

| Gene sets | NOM p value | FDR q value |
| --- | --- | --- |
| **C2 collection: curated gene sets** | | |
| **Enriched in beta–cell samples from T2D subjects** | | |
| ZUCCHI_EPITHELIAL_DN | 0.000 | 0.128 |
| HSA04610_COMPLEMENT_AND_COAGULATION_CASCADES | 0.000 | 0.247 |
| **Enriched in beta–cell samples from control subjects** |  |  |
| HDACI_COLON_CLUSTER5 | 0.000 | 0.109 |
|  |  |  |
|  |  |  |
| **C5 collection: GO gene sets** | | |
| **Biological Process Ontology Gene sets** | | |
| **Enriched in beta–cell samples from T2D subjects** | | |
| Viral processes |  |  |
| VIRAL_INFECTIOUS_CYCLE | 0.000 | 0.072 |
| VIRAL_REPRODUCTIVE_PROCESS | 0.000 | 0.080 |
| VIRAL_GENOME_REPLICATION | 0.018 | 0.105 |
| VIRAL_REPRODUCTION | 0.000 | 0.108 |
|  |  |  |
| JNK activity |  |  |
| REGULATION_OF_JNK_ACTIVITY | 0.000 | 0.088 |
| POSITIVE_REGULATION_OF_JNK_ACTIVITY | 0.000 | 0.104 |
| ACTIVATION_OF_JNK_ACTIVITY | 0.000 | 0.114 |
|  |  |  |
| Response to stimulus |  |  |
| REGULATION_OF_RESPONSE_TO_STIMULUS | 0.000 | 0.110 |
| POSITIVE_REGULATION_OF_RESPONSE_TO_STIMULUS | 0.000 | 0.195 |
|  |  |  |
| Immune response |  |  |
| ACTIVATION_OF_IMMUNE_RESPONSE | 0.000 | 0.134 |
| HUMORAL_IMMUNE_RESPONSE | 0.018 | 0.204 |
|  |  |  |
| Hormone secretion |  |  |
| HORMONE_SECRETION | 0.000 | 0.119 |
|  |  |  |
| Cell-cell adhesion |  |  |
| CALCIUM_INDEPENDENT_CELL_CELL_ADHESION | 0.019 | 0.217 |
|  |  |  |
| **Enriched in beta–cell samples from control subjects** | | |
| Tissue remodeling |  |  |
| BONE_REMODELING | 0.000 | 0.008 |
| TISSUE_REMODELING | 0.000 | 0.020 |
|  |  |  |
| Fatty acid oxidation |  |  |
| FATTY_ACID_OXIDATION | 0.000 | 0.020 |
|  |  |  |
| Nucleotide metabolism |  |  |
| NUCLEOTIDE_BIOSYNTHETIC_PROCESS | 0.000 | 0.106 |
| NUCLEOBASE_NUCLEOSIDE_AND_NUCLEOTIDE_METABOLIC_PROCESS | 0.021 | 0.165 |
|  |  |  |
| **Molecular Function Ontology Gene sets** | | |
| **Enriched in beta–cell samples from T2D subjects** | | |
| Metabolic activity |  |  |
| PROTEASE_INHIBITOR_ACTIVITY | 0.000 | 0.097 |
| ATP_DEPENDENT_RNA_HELICASE_ACTIVITY | 0.020 | 0.105 |
| SULFURIC_ESTER_HYDROLASE_ACTIVITY | 0.039 | 0.199 |
| METALLOPEPTIDASE_ACTIVITY | 0.000 | 0.216 |
|  |  |  |
| Chemokine and G protein coupled receptor binding activity |  |  |
| G_PROTEIN_COUPLED_RECEPTOR_BINDING | 0.000 | 0.115 |
| CHEMOKINE_RECEPTOR_BINDING | 0.000 | 0.143 |
| CHEMOKINE_ACTIVITY | 0.000 | 0.214 |
|  | | |
| **Cellular Component Ontology Gene sets** | | |
| **Enriched in beta–cell samples from T2D subjects** | | |
| Vacuole localization |  |  |
| LYTIC_VACUOLE | 0.000 | 0.058 |
| LYSOSOME | 0.000 | 0.116 |
| VACUOLE | 0.000 | 0.226 |

Gene sets differentially regulated in samples of T2D subjects and control subjects. The nominal p value < 0.01 at multiple hypothesis testing false discovery rate (FDR) ≤ 25% was used as parameter to detect the differentially regulated probe sets.
